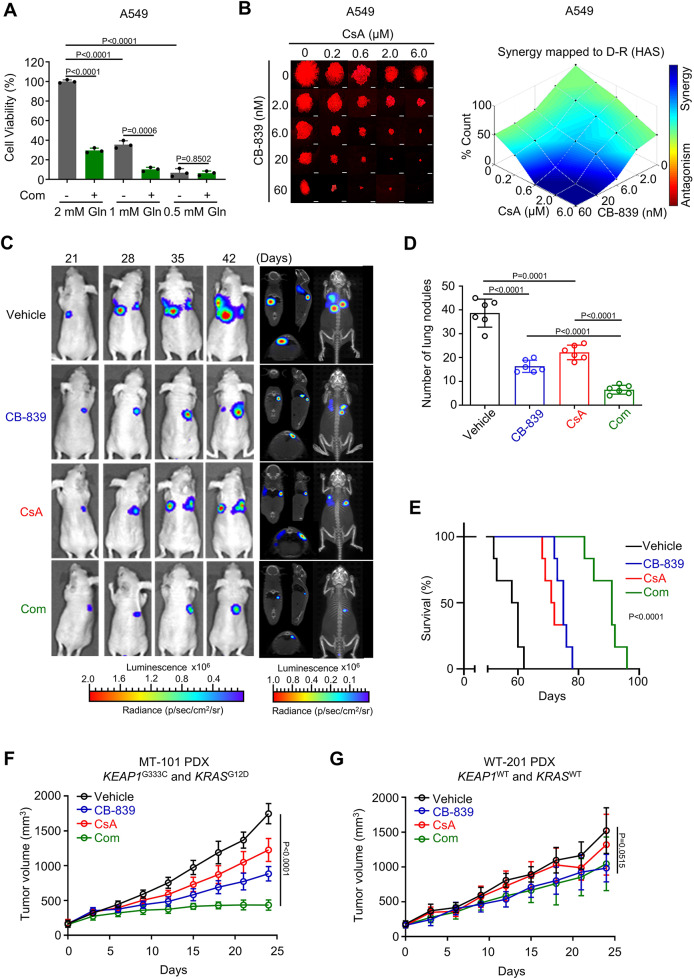# Author Correction: PPIA dictates NRF2 stability to promote lung cancer progression

**DOI:** 10.1038/s41467-025-67497-8

**Published:** 2025-12-15

**Authors:** Weiqiang Lu, Jiayan Cui, Wanyan Wang, Qian Hu, Yun Xue, Xi Liu, Ting Gong, Yiping Lu, Hui Ma, Xinyu Yang, Bo Feng, Qi Wang, Naixia Zhang, Yechun Xu, Mingyao Liu, Ruth Nussinov, Feixiong Cheng, Hongbin Ji, Jin Huang

**Affiliations:** 1 Shanghai Frontiers Science Center of Optogenetic Techniques for Cell Metabolism, Shanghai Key Laboratory of New Drug Design, School of Pharmacy, East China University of Science and Technology, Shanghai, China; 2 Shanghai Key Laboratory of Regulatory Biology, Institute of Biomedical Sciences and School of Life Sciences, East China Normal University, Shanghai, China; 3 State Key Laboratory of Cell Biology, Shanghai Institute of Biochemistry and Cell Biology, Center for Excellence in Molecular Cell Science, Chinese Academy of Sciences, Shanghai, China; 4 School of Life Science, Hangzhou Institute for Advanced Study, University of Chinese Academy of Sciences, Hangzhou, China; 5 Department of General Surgery, Ruijin Hospital, Shanghai Jiao Tong University School of Medicine, Shanghai, China; 6 Key Laboratory of Early Prevention and Treatment for Regional High Frequency Tumor, Ministry of Education, Nanning, China; 7 Guangxi Medical University Cancer Hospital, Nanning, China; 8 Shanghai Institute of Materia Medica, Chinese Academy of Sciences, Shanghai, China; 9 Computational Structural Biology Section, Basic Science Program, Frederick National Laboratory for Cancer Research, National Cancer Institute at Frederick, Frederick, USA; 10 Department of Human Molecular Genetics and Biochemistry, Sackler School of Medicine, Tel Aviv University, Tel Aviv, Israel; 11 Genomic Medicine Institute, Lerner Research Institute, Cleveland Clinic, Cleveland, USA

**Keywords:** Non-small-cell lung cancer, Targeted therapies, Cancer metabolism

Correction to: *Nature Communications* 10.1038/s41467-024-48364-4, published online 03 June 2024

In the version of the article initially published, in Fig. 6c, the bioluminescence image of CB-839 group at day 35 was incorrect. This error has now been corrected in both the HTML and PDF versions of the article as seen in Fig. 1 below.

Fig. 1 Original and corrected Fig. 6c

Original Fig. 6c
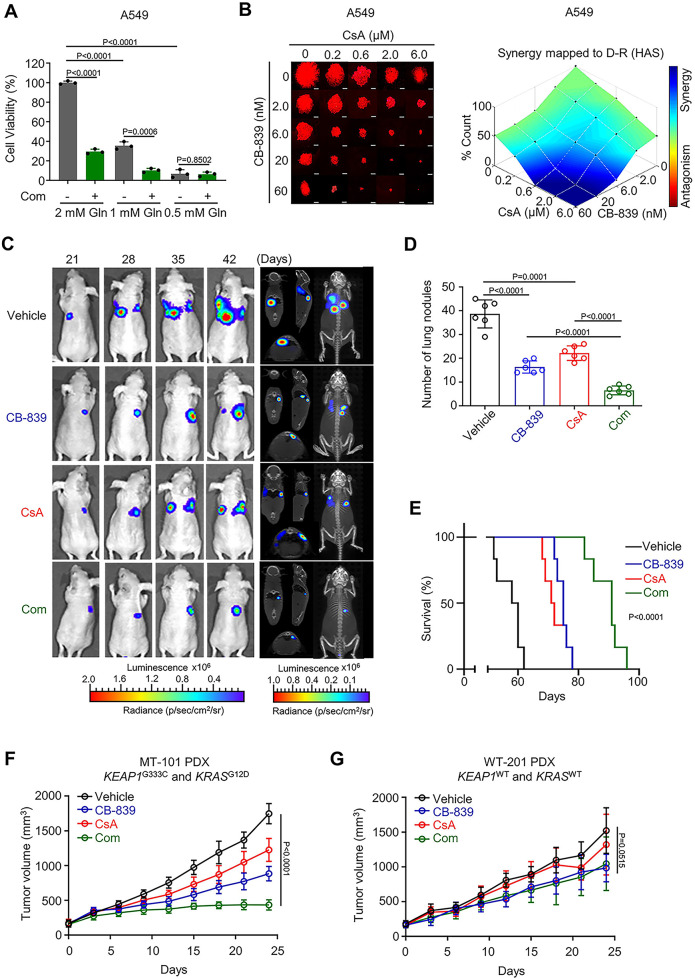


Corrected Fig. 6c