# Supplementary material for: PPIA dictates NRF2 stability to promote lung cancer progression
Source: Nat Commun. 2024 Jun 3;15:4703. doi: 10.1038/s41467-024-48364-4 (PMC11148020; doi:10.1038/s41467-024-48364-4)
Supplement: Supplementary file 1 — Supplementary Information [file 41467_2024_48364_MOESM1_ESM.docx]

**Supplementary Information**

**PPIA dictates NRF2 stability to promote lung cancer progression**

Weiqiang Lu^1,2,*,#^, Jiayan Cui^1,#^, Wanyan Wang^1,#^, Qian Hu^1,#^, Yun Xue^3,#^, Xi Liu^1^, Ting Gong^1^, Yiping Lu^1^, Hui Ma^1^, Xinyu Yang^2^, Bo Feng^4^, Qi Wang^5^, Naixia Zhang^6^, Yechun Xu^6^, Mingyao Liu^2^, Ruth Nussinov^7^, Feixiong Cheng^8^, Hongbin Ji^3^, Jin Huang^1,*^

^1^Shanghai Frontiers Science Center of Optogenetic Techniques for Cell Metabolism, Shanghai Key Laboratory of New Drug Design, School of Pharmacy, East China University of Science and Technology, Shanghai, China.

^2^Shanghai Key Laboratory of Regulatory Biology, Institute of Biomedical Sciences and School of Life Sciences, East China Normal University, Shanghai, China.

^3^State Key Laboratory of Cell Biology, Shanghai Institute of Biochemistry and Cell Biology, Center for Excellence in Molecular Cell Science, Chinese Academy of Sciences, Shanghai, China; School of Life Science, Hangzhou Institute for Advanced Study, University of Chinese Academy of Sciences, Hangzhou, China.

^4^Department of General Surgery, Ruijin Hospital, Shanghai Jiao Tong University School of Medicine, Shanghai, China.

^5^Key Laboratory of Early Prevention and Treatment for Regional High Frequency Tumor, Ministry of Education, Nanning, China; Guangxi Medical University Cancer Hospital, Nanning, China.

^6^Shanghai Institute of Materia Medica, Chinese Academy of Sciences, Shanghai, China.

^7^Computational Structural Biology Section, Basic Science Program, Frederick National Laboratory for Cancer Research, National Cancer Institute at Frederick, Frederick, U.S.A; Department of Human Molecular Genetics and Biochemistry, Sackler School of Medicine, Tel Aviv University, Israel.

^8^Genomic Medicine Institute, Lerner Research Institute, Cleveland Clinic, Cleveland, U.S.A.

^#^These authors contributed equally to this work.

^*^Corresponding authors.

**Supplementary Figure 1**

**
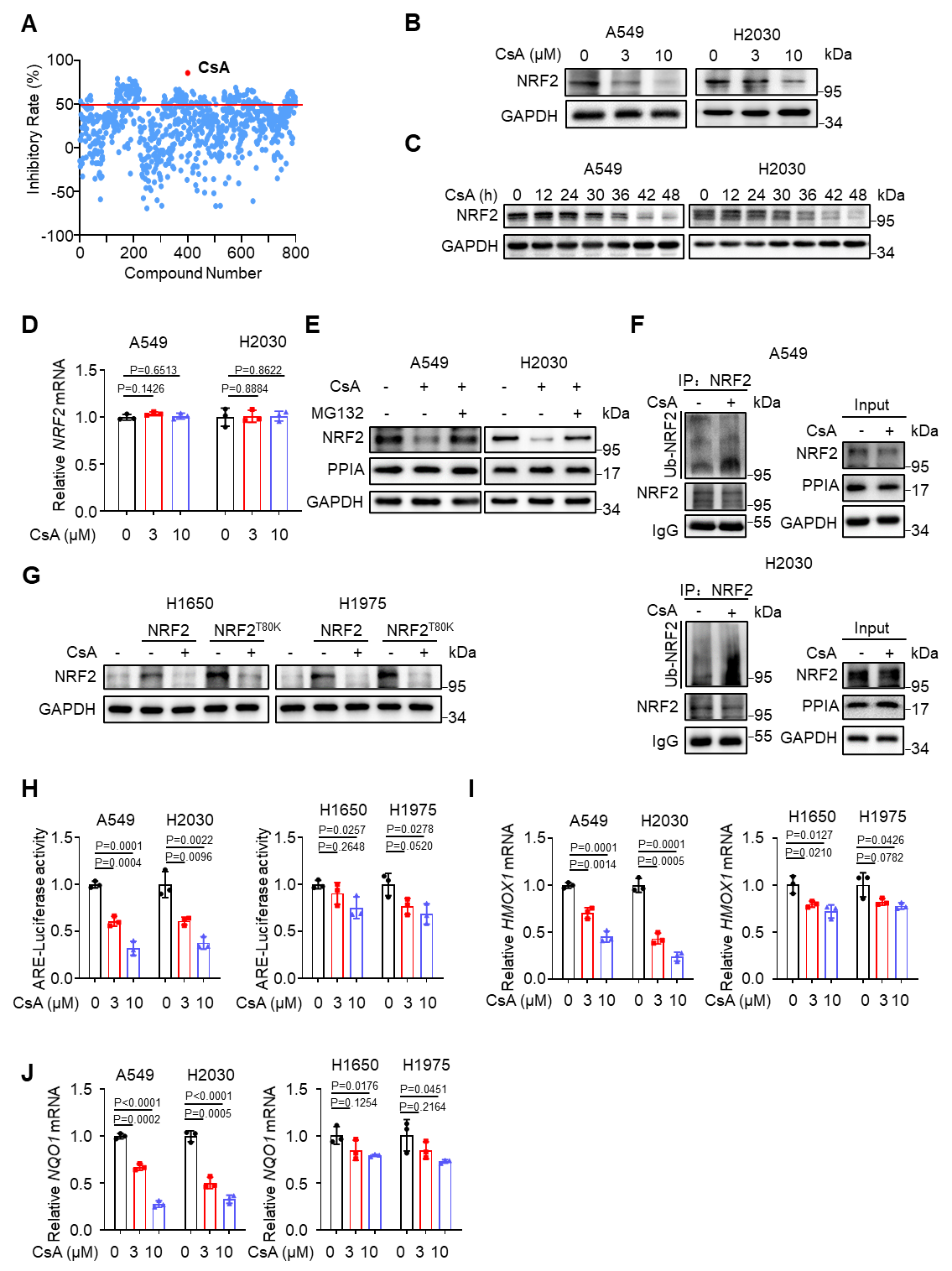
**

**Supplementary Figure 1. Related to Figure 1**

(A) RIG-NRF2 based screening of NRF2 protein stability against an in-house library of 805 compounds (including clinical or approved drugs) in A549 cells.

(B) Representative immunoblot analysis of NRF2 levels in A549 and H2030 cells treated with CsA for 48 h.

(C) Representative immunoblot analysis of NRF2 levels upon a time course of CsA treatment. A549 and H2030 cells were treated with 10 μM CsA for 0, 12, 24, 30, 36, 42 or 48 h and then subjected to immunoblot analysis.

(D) Q-PCR analysis of gene expression of *NRF2* in A549 and H2030 cells treated with CsA for 48 h.

(E) Representative immunoblot analysis of NRF2 levels in A549 and H2030 cells treated with CsA (10 μM) in the presence or absence of proteasome inhibitor MG132.

(F) Immunoprecipitation of NRF2 followed by immunoblot analysis with anti-ubiquitin antibody detected the NRF2 ubiquitination in A549 and H2030 cells treated with CsA (10 μM).

(G) Representative immunoblot analysis of NRF2 levels in H1650 and H1975 cells upon NRF2^WT^ or NRF2^T80K^ overexpression with or without CsA treatment (10 μM).

(H) ARE reporter activity of pGL4.37-ARE-luc in A549, H2030, H1650 and H1975 cells treated with CsA (3, 10 μM) for 48 h.

(I-J) Q-PCR analysis of gene expression of *HMOX1* (I), and *NQO1* (J) in A549, H2030, H1650 and H1975 cells treated with CsA for 48 h.

The results of panels (B, C, E, F, G) are representative of three independent experiments. D, H-J Data are presented as mean ± SD of three independent experiments. P values were analyzed using Two-tailed unpaired Student’s t-test, P < 0.05 was considered statistically significant. Source data are provided as a Source Data file.

**Supplementary Figure 2**


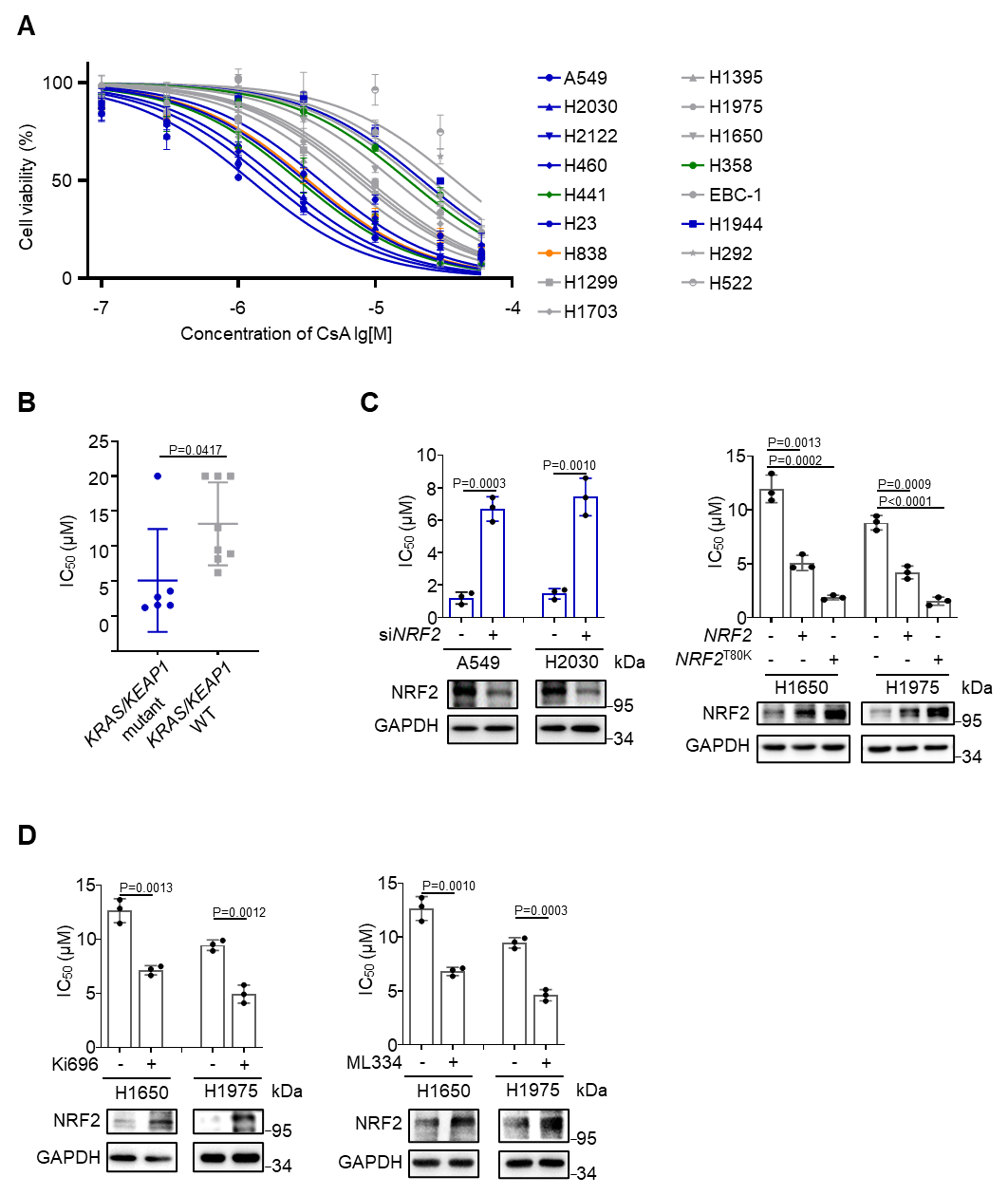


**Supplementary Figure 2. Related to Figure 1**

(A) The dose-response curves of CsA against 17 NSCLC cell lines related to **Figure 1E**. Cell viability was determined by using MTT.

(B) A dot plot showing the IC_50_ values of CsA against cell lines with *KRAS* and *KEAP1* co-mutations (n = 6 cell lines) and against cell lines with *KRAS* and *KEAP1* WT (n = 8 cell lines) related to **Supplementary Table 1**. P value was analyzed using Two-tailed unpaired Student’s t-test.

(C) IC_50_ values of CsA in *KRAS/KEAP1* co-mutant cell lines (A549 and H2030) following *NRF2* knockdown and *KRAS/KEAP1* WT cell lines (H1650 and H1975) following *NRF2* or *NRF2^T80K^* overexpression.

(D) IC_50_ values of CsA in H1650 and H1975 (KRAS/KEAP1 WT cell lines) upon Ki696 (1 μM, 8 h) or ML334 (50 μM, 16 h) treatment.

(A) and (C-D) represent mean ± SD of three biologically independent experiments. P values were analyzed using Two-tailed unpaired Student’s t-test, P < 0.05 was considered statistically significant. Source data are provided as a Source Data file.

**Supplementary Figure 3**

**
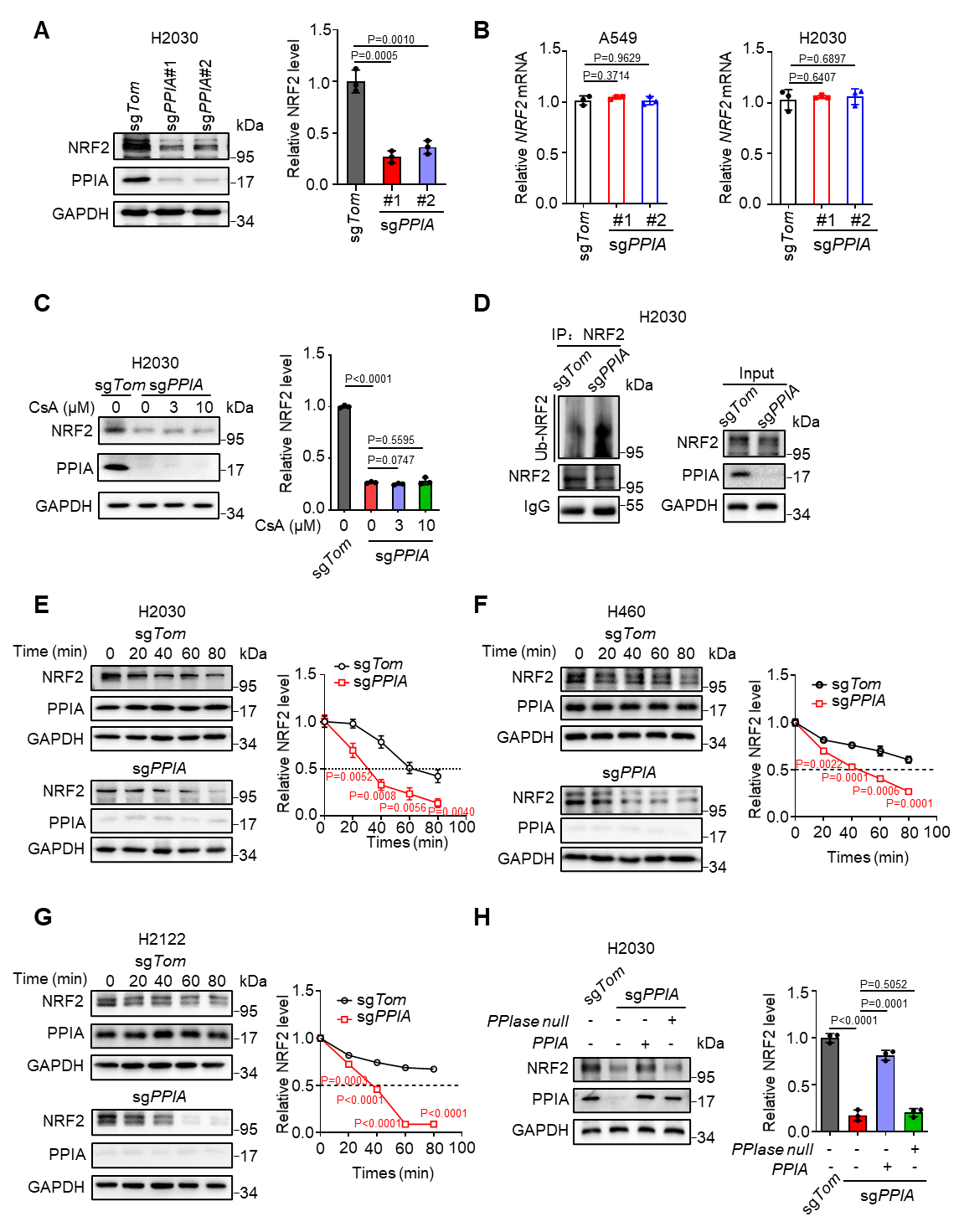
**

**Supplementary Figure 3. Related to Figure 2**

(A) Representative immunoblot analysis of NRF2 levels in *PPIA*-WT or *PPIA*-KO H2030 cells. Quantitative results were shown in the right panel.

(B) Q-PCR analysis of *NRF2* expression in *PPIA*-WT or *PPIA*-KO A549 and H2030 cells.

(C) Representative immunoblot analysis of NRF2 levels in *PPIA*-WT or *PPIA-*KO H2030 cells treated with CsA (0, 3, 10 μM) for 48 h. Quantitative results were shown in the right panel.

(D) Immunoprecipitation of NRF2 followed by immunoblot analysis with anti-ubiquitin antibody detected the NRF2 ubiquitination in *PPIA*-WT and *PPIA*-KO H2030 cells.

(E-G) Cycloheximide (CHX) chase assay of NRF2 protein stability in *PPIA*-WT or *PPIA*-KO H2030 (E), H460 (F) and H2122 (G) cells. Cells were treated with CHX (100 μg/mL) at the indicated time points and then subjected to immunoblot. Quantitative results were shown in the right panel.

(H) Representative immunoblot demonstrates that *PPIA* WT, but not *PPIA* catalytically dead PPIA variant (PPIA^R55A&F60A^), can restore the NRF2 level in *PPIA*-KO H2030 cells. Quantitative results were shown in the right panel.

The results of panels (A, C-H) are representative of three independent experiments. (A-C, E-H) represent mean ± SD of three independent experiments. P values were analyzed using Two-tailed unpaired Student’s t-test, P < 0.05 was considered statistically significant. Source data are provided as a Source Data file.

**Supplementary Figure 4**


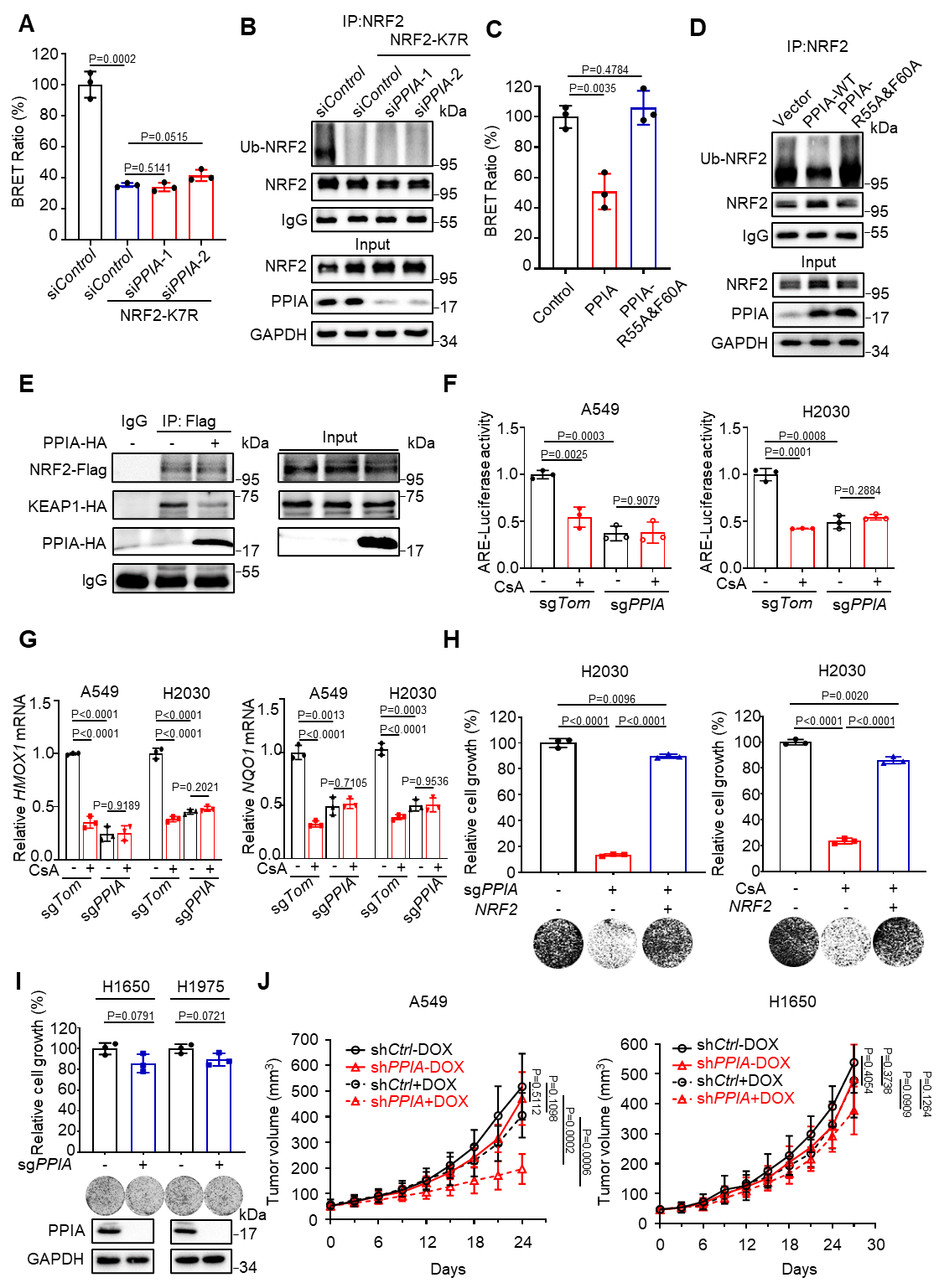


**Supplementary Figure 4. Related to Figure 2**

(A) BRET signal of HEK293T cells transfected with HaloTag^®^-Ubiquitin and NRF2-Nanoluc or NRF2-K7R-Nanoluc (seven lysine residues between DLG and ETGE motif in Neh2 domain of NRF2 were replaced by arginine residues) following si*PPIA* treatment or not.

(B) Immunoprecipitation of NRF2 followed by immunoblot analysis with anti-ubiquitin antibody detected the NRF2 ubiquitination in HEK293T following si*PPIA* treatment or not.

(C) BRET signal of HEK293T cells transfected with NRF2-Nanoluc and HaloTag^®^-Ubiquitin following overexpression of PPIA-WT or PPIA-R55A&F60A.

(D) Immunoprecipitation of NRF2 followed by immunoblot analysis with anti-ubiquitin antibody detected the NRF2 ubiquitination in HEK293T following overexpression of PPIA-WT or PPIA-R55A&F60A.

(E) Co-immunoprecipitation analysis of Flag-tagged NRF2 with PPIA and KEAP1 in HEK293T cells.

(F) ARE-driven luciferase activity in *PPIA*-WT or *PPIA*-KO A549 and H2030 cells treated with CsA (10 μM) for 48 h.

(G) Q-PCR analysis of NRF2 target genes *HMOX1* and *NQO1* in *PPIA*-WT or *PPIA*-KO A549 and H2030 cells treated with CsA (10 μM) for 48 h.

(H) Relative cell growth of H2030 upon *PPIA* KO or CsA treatment in the presence or absence of *NRF2* overexpression. Quantitative results were shown in upper panel and colony images were presented in down panel.

(I) Relative cell growth of H1650 or H1975 upon *PPIA* KO and representative immunoblot analysis of PPIA protein level in WT or *PPIA*-KO H1650 and H1975 cells were represented, respectively. Quantitative results were shown in up panel, colony images were shown in middle panel and representative immunoblot results were presented in down panel.

(J) A549 and H1650 with inducible sh*Ctrl* or sh*PPIA* were used to generate subcutaneous tumors in mice. When tumor volumes reached approximately 50 mm^3^, doxycycline (20 mg/kg) was orally administrated to mice every other day. Tumor volume was measured every three days using slide calipers (n = 6 mice per group).

The results of panels (B, D, E, I) are representative of three independent experiments. (A), (C) and (F-I) represent mean ± SD of three independent experiments. P values were analyzed using Two-tailed unpaired Student’s t-test, P < 0.05 was considered statistically significant. Source data are provided as a Source Data file.

**Supplementary Figure 5**

**
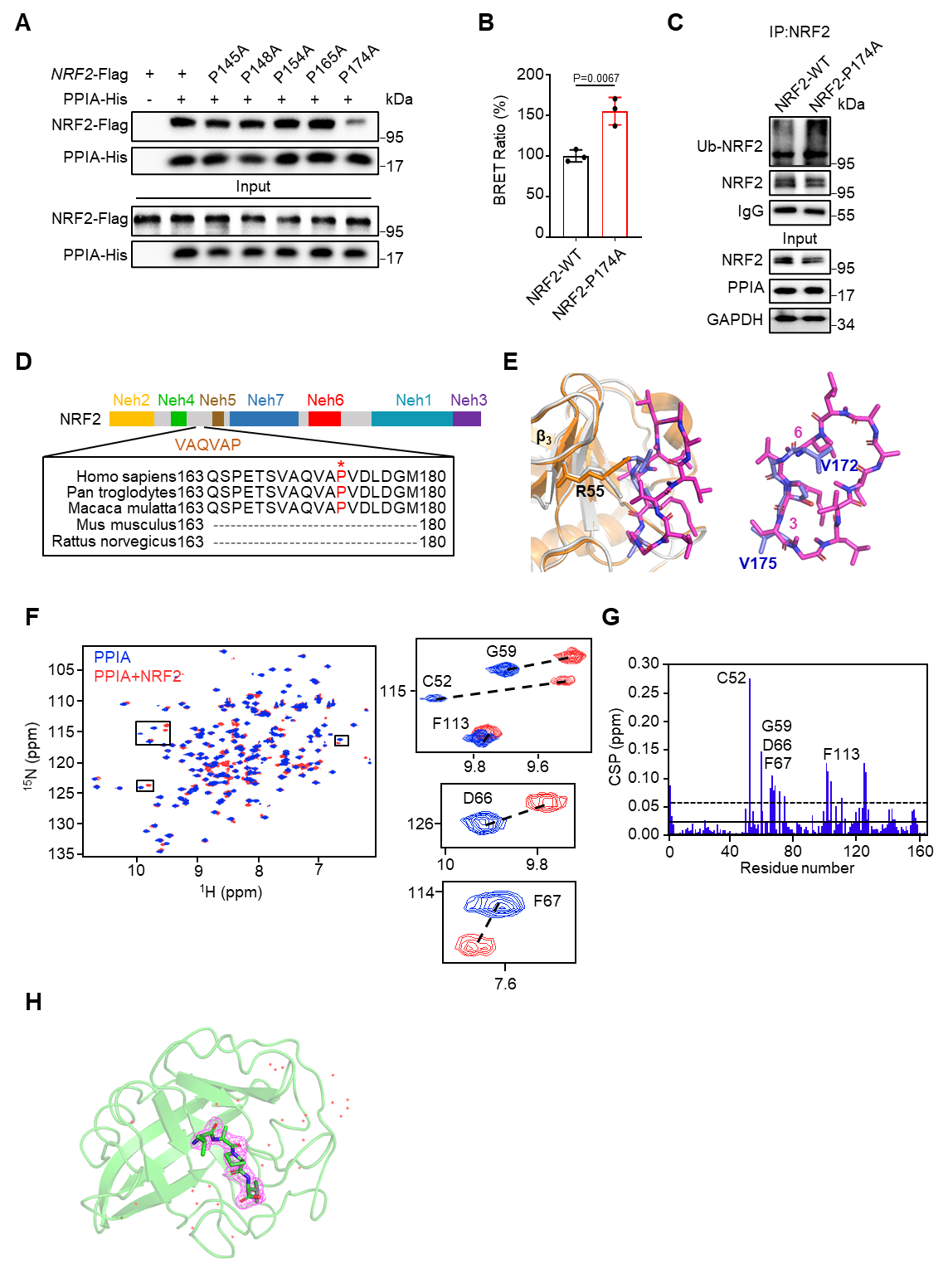
**

**Supplementary Figure 5. Related to Figure 3**

(A) Pull-down analysis of PPIA and *NRF2* (WT or mutants including P145A, P148A, P154A, P165A, and P174A).

(B) BRET signal of HEK293T cells transfected with HaloTag^®^-Ubiquitin and NRF2-Nanoluc or NRF2-P174A-Nanoluc.

(C) Immunoprecipitation of NRF2 followed by immunoblot analysis with anti-ubiquitin antibody detected the NRF2 ubiquitination in HEK293T following overexpression of NRF2 or NRF2-P174A.

(D) Alignment of the 163-180 regions of NRF2 of various species.

(E) Crystal structure comparison of PPIA-CsA complex (PDB ID: 1CWA) and PPIA-PBM fragment (PDB ID: 8HZ8). In the PPIA-CsA complex, PPIA is shown as orange cartoon and CsA is displayed as magenta sticks. In PPIA-PBM fragment complex, PPIA is shown as gray cartoon and PBM fragment is displayed as slate sticks.

(F) Superposition of the [^1^H, ^15^N] HSQC spectra of PPIA without (blue) or with the addition of 5-fold molar excess of NRF2 PBM fragment (red). Selected ^1^H-^15^N-HSQC spectra regions are expanded to view representative residues which undergo significant chemical shift perturbations upon the binding of NRF2 PBM fragment.

(G) Chemical shift changes in labeled PPIA upon addition of NRF2 PBM fragment in (F).

(H) The 2Fo−Fc density map of the VAPV bound to PPIA. The density map is contoured at 1.0 σ.

The results of panels (A, C) are representative of three independent experiments. (B) represents mean ± SD of three independent experiments. P value was analyzed by Two-tailed unpaired Student’s t-test. Source data are provided as a Source Data file.

**Supplementary Figure 6**

**
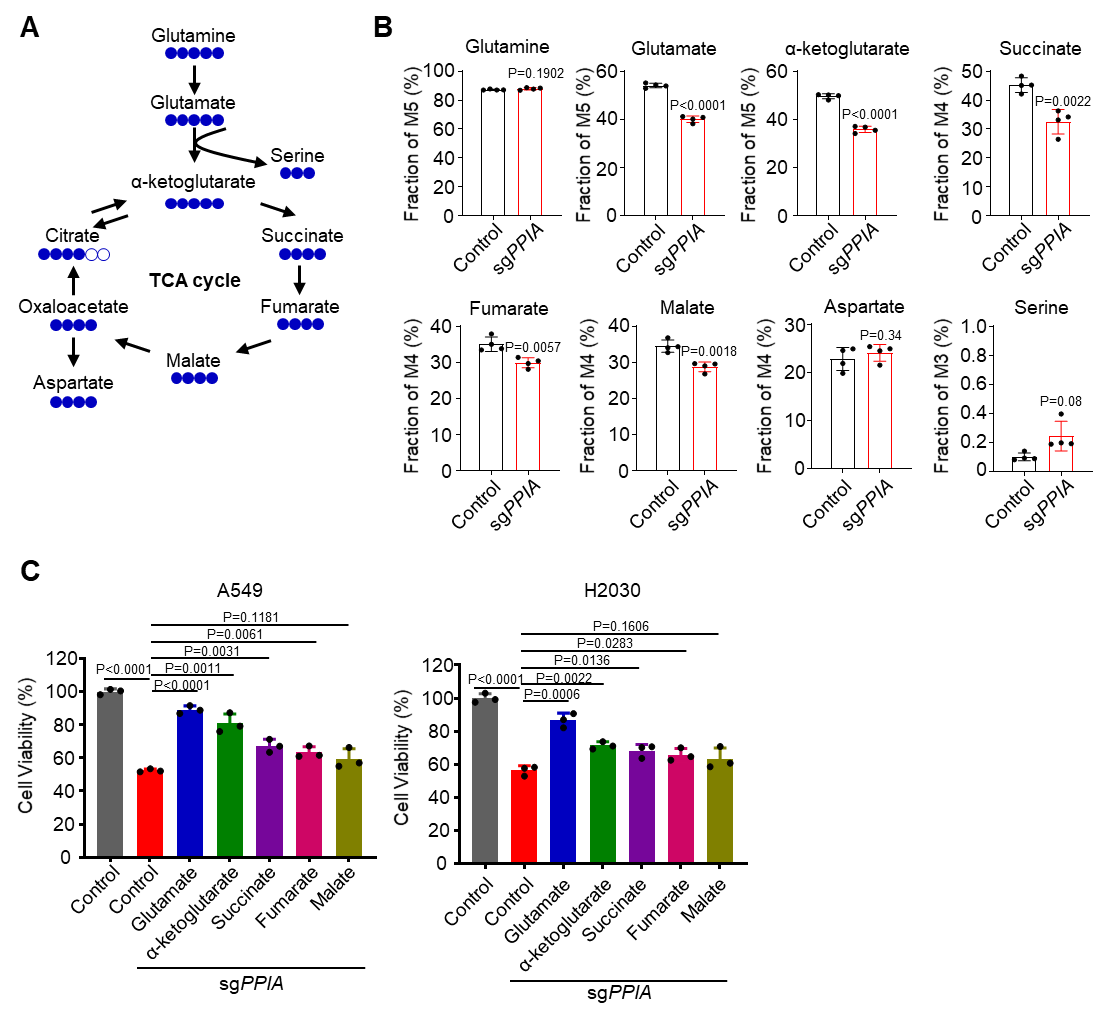
**

**Supplementary Figure 6. Related to Figure 4**

(A) Schematic of ^13^C isotopomer patterns in TCA cycle intermediates arising from glutamine metabolism. Blue dots represent ^13^C atoms derived from ^13^C-glutamine.

(B) Contribution of ^13^C-glutamine derived TCA metabolites in A549 cells following *PPIA* KO.

(C) Relative cell viability of *PPIA*-KO A549 or *PPIA*-KO H2030 supplemented with glutamate (2 mM), α-ketoglutarate (0.5 mM), succinate (0.5 mM), fumarate (0.5 mM) and malate (0.5 mM). Data is presented as relative to the proliferation of *PPIA* WT A549 or *PPIA* WT H2030.

(B) represents mean ± SD of four independent experiments. (C) represents mean ± SD of three independent experiments. P values were analyzed using Two-tailed unpaired Student’s t-test, P < 0.05 was considered statistically significant. Source data are provided as a Source Data file.

**Supplementary Figure 7**


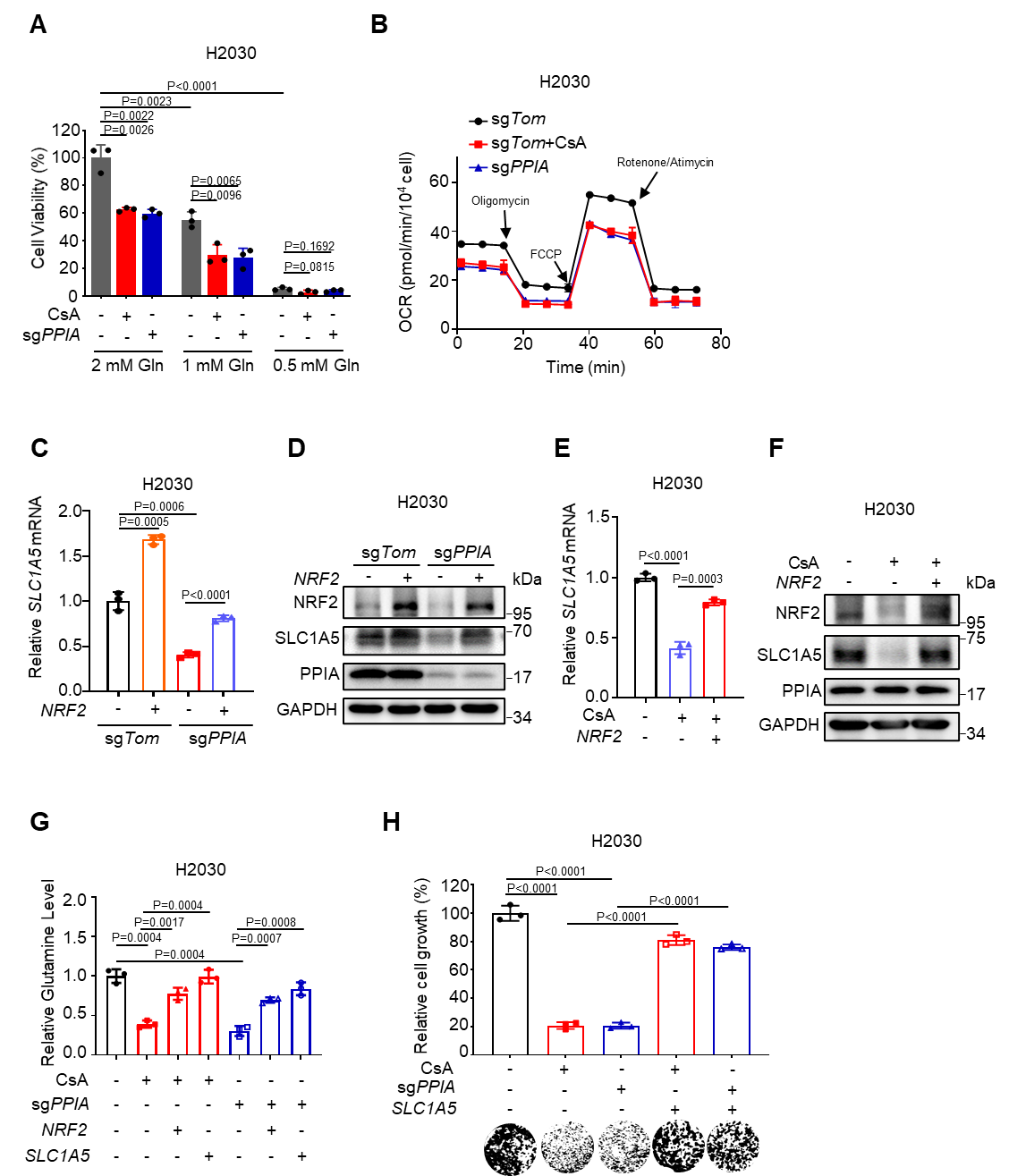


**Supplementary Figure 7. Related to Figure 4**

(A) H2030 cells were subjected to CsA (10 μM) treatment or *PPIA* KO in the presence of glutamine (2, 1, 0.5 mM) and cell viability was measured by MTT assay. Gln, glutamine.

(B) Oxygen consumption rate (OCR) plotted over time in H2030 cells following CsA (10 μM) treatment or *PPIA* KO.

(C-D) Q-PCR (C) and immunoblot (D) analysis of SLC1A5 in *PPIA*-WT or *PPIA*-KO H2030 cells following *NRF2* overexpression.

(E-F) Q-PCR (E) and immunoblot (F) results of SLC1A5 in H2030 cells treated with CsA (10 μM for 48 h) with or without *NRF2* overexpression.

(G) Relative glutamine levels in H2030 cells upon CsA (10 μM) treatment or *PPIA* KO following *SLC1A5* or *NRF2* overexpression.

(H) H2030 cells upon CsA (10 μM) treatment or *PPIA* KO following *SLC1A5* overexpression were subjected to colony formation. Quantitative results were shown in upper panel and colony images were presented in lower panel.

The results of panels (D, F) are representative of three independent experiments. (A-C), (E), and (G-H) represent mean ± SD of three independent experiments. P values were analyzed using Two-tailed unpaired Student’s t-test, P < 0.05 was considered statistically significant. Source data are provided as a Source Data file.

**Supplementary Figure 8**

**
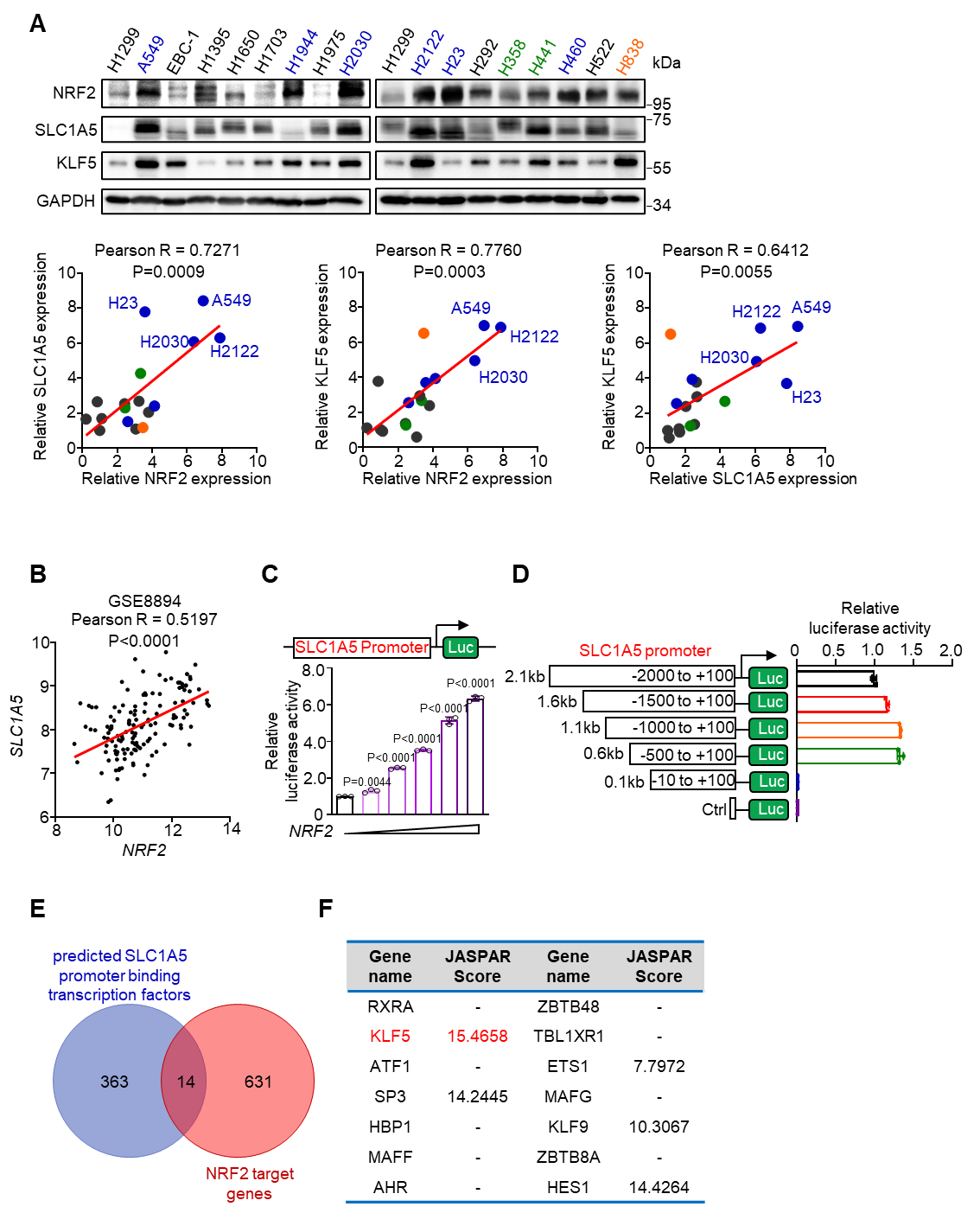
**

**Supplementary Figure 8. Related to Figure 5**

(A) Representative immunoblot analysis of NRF2, SLC1A5 and KLF5 protein levels in NSCLC cell lines was shown in the upper panel. Scatter plots showing the correlation of NRF2 and SLC1A5, NRF2 and KLF5, SLC1A5 and KLF5 protein levels were shown in the lower panel.

(B) Correlation analysis of *NRF2* and *SLC1A5* gene expression in clinical NSCLC tumor samples. The data are derived from public dataset (GSE8894) and analyzed in PrognoScan.

(C) pGL3-Luc vector containing human *SLC1A5* promoter (-2,000 to +10 bp) and pCDNA3.1-Flag-NRF2 were co-transfected into HEK293T cells with a ratio of 1:0.5 to 1:8 and luciferase activity was determined.

(D) pGL3-Luc vector containing distinct regions of human *SLC1A5* promoter and pCDNA3.1-Flag-NRF2 were co-transfected into HEK293T cells and luciferase activity was measured.

(E) Venn diagram showed overlap between 377 predicted SLC1A5 promoter binding transcription factors (https://www.genecards.org) and 645 genes potential NRF2 target genes revealed by ChIP-Seq.

(F) The binding score of *SLC1A5* promoter predicted by JASPAR of 14 overlapped genes in (E).

The result of (A) is representative of three independent experiments. (C-D) represent mean ± SD of three independent experiments. P values were analyzed using Two-tailed unpaired Student’s t-test, P < 0.05 was considered statistically significant. Source data are provided as a Source Data file.

**Supplementary Figure 9**


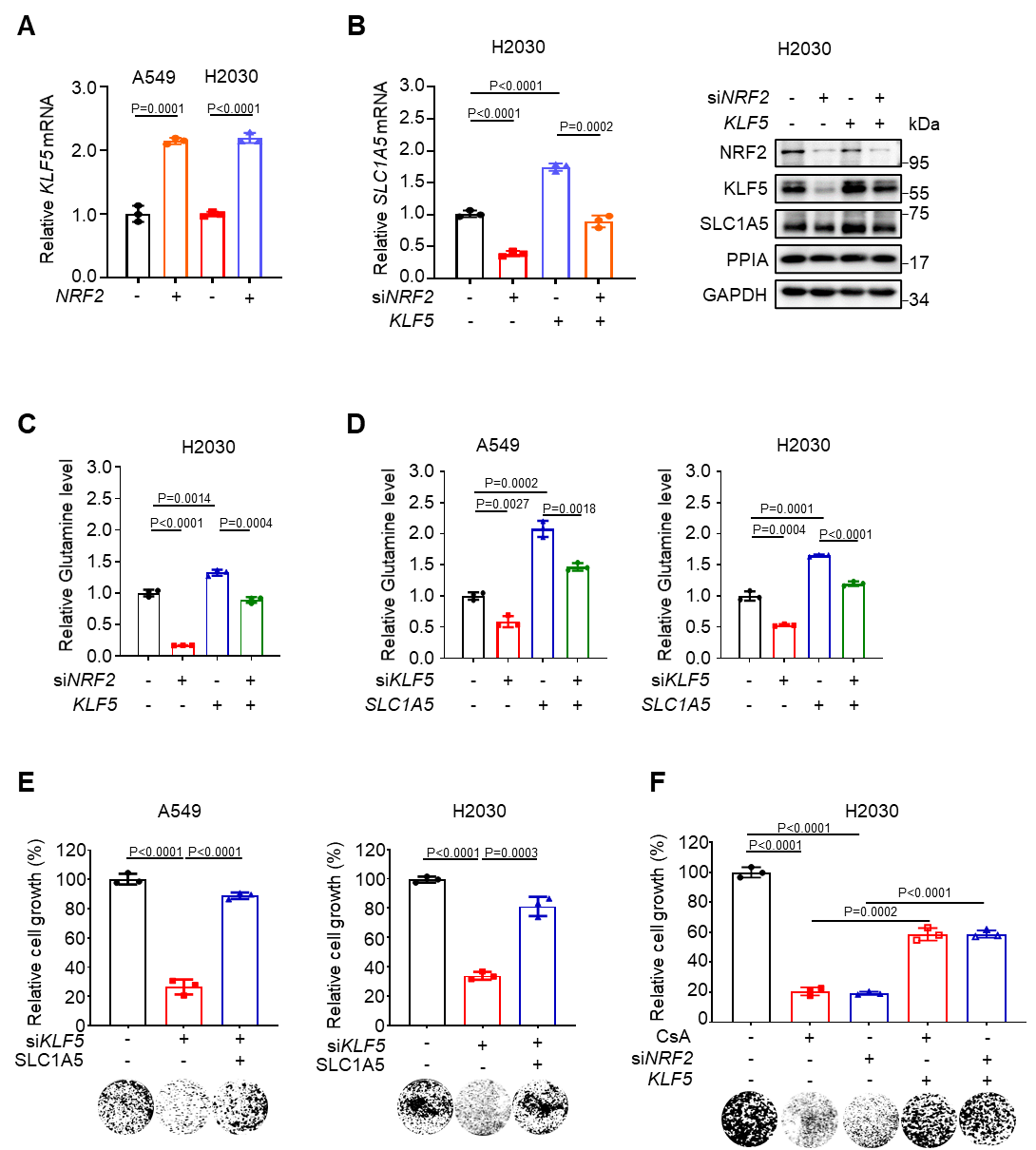


**Supplementary Figure 9. Related to Figure 5**

(A) Overexpression of NRF2 elevated the mRNA levels of *KLF5* as revealed by Q-PCR in A549 and H2030 cells.

(B) Q-PCR and immunoblot results of SLC1A5 in H2030 cells treated with si*NRF2* in the presence or absence of *KLF5* overexpression.

(C) Relative glutamine levels in H2030 cells following si*NRF2* treatment in the presence or absence of *KLF5* overexpression.

(D) Relative glutamine levels in A549 and H2030 cells following si*KLF5* treatment with or without SLC1A5 overexpression.

(E) Colony formation of A549 and H2030 cells following si*KLF5* treatment with or without SLC1A5 overexpression. Quantitative results were shown in upper panel and representative colony image was presented in lower panel.

(F) Colony formation of H2030 cells treated with CsA or si*NRF2* in the presence or absence of *KLF5* overexpression. Quantitative results were shown in upper panel and colony images were presented in lower panel.

Immunoblots in (B) is representative of three independent experiments. (A-F) represent mean ± SD of three independent experiments. P values were analyzed using Two-tailed unpaired Student’s t-test, P < 0.05 was considered statistically significant. Source data are provided as a Source Data file.

**Supplementary Figure 10**

**
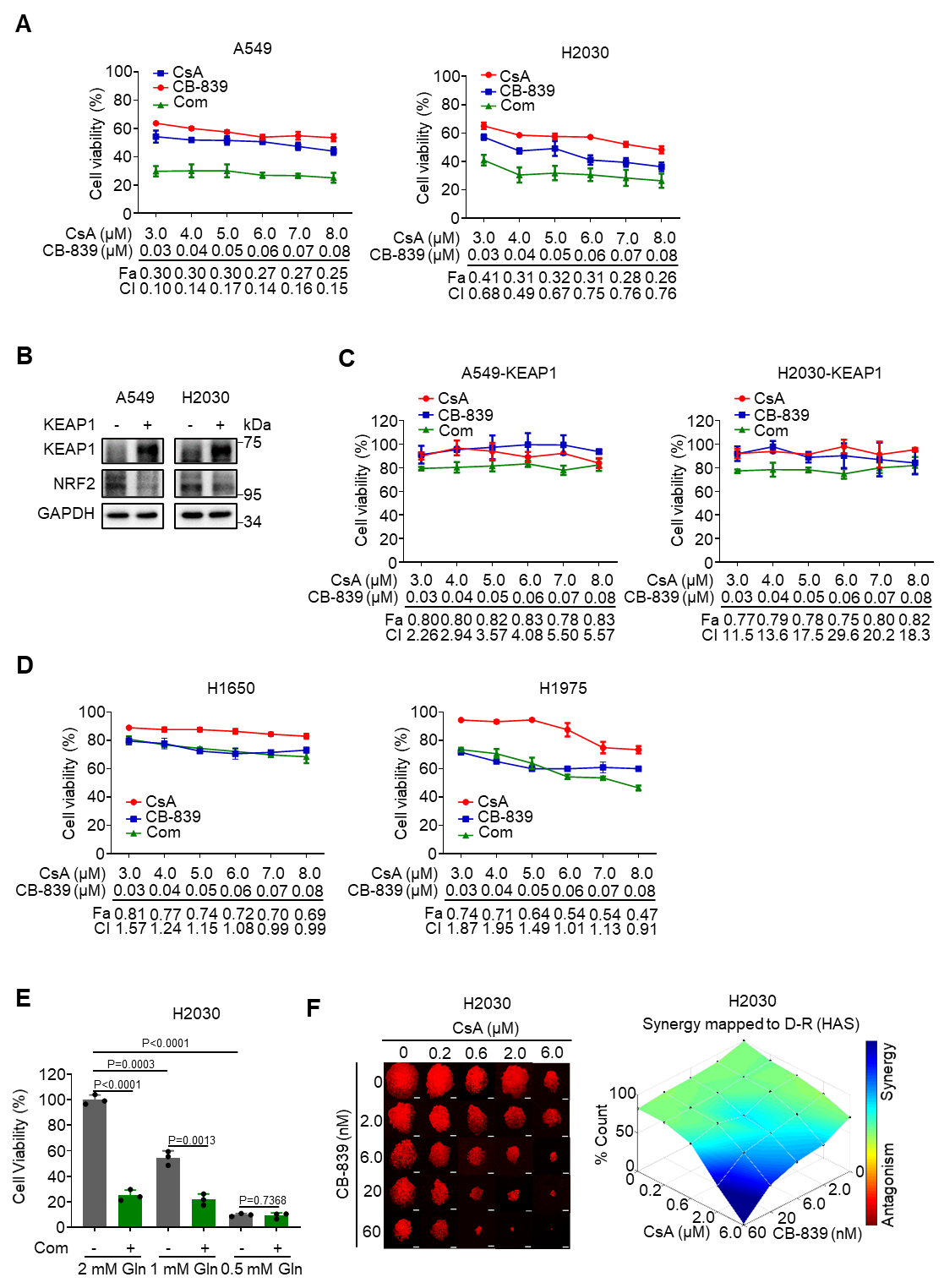
**

**Supplementary Figure 10. Related to Figure 6**

(A) A549 and H2030 were treated with vehicle, CsA, CB-839, or their combination (n = 3 independent experiments). Cell viability was determined by using MTT assay, and combination index (CI) were calculated via the Chou-Talalay equation. CI values <1, =1, and >1 indicate synergism, additive, and antagonism, respectively.

(B) Representative immunoblot analysis of KEAP1 and NRF2 levels in A549 and H2030 cells upon *KEAP1* overexpression.

(C) A549 and H2030 with *KEAP1* overexpression were treated with vehicle, CsA, CB-839, or their combination (n = 3 independent experiments). Cell viability was determined by using MTT assay, and combination index (CI) were calculated via the Chou-Talalay equation. CI values <1, =1, and >1 indicate synergism, additive, and antagonism, respectively.

(D) H1650 and H1975 were treated with vehicle, CsA, CB-839, or their combination (n = 3 independent experiments). Cell viability was determined by using MTT assay, and combination index (CI) were calculated via the Chou-Talalay equation. CI values <1, =1, and >1 indicate synergism, additive, and antagonism, respectively.

(E) Cell viability of H2030 cells treated with CsA (8 μM) and CB-839 (0.08 μM) combination (Com) in presence of 2 mM, 1 mM or 0.5 mM glutamine (Gln).

(F) 3D clonogenic assay of H2030-mCherry cells treated with vehicle, CsA, CB-839, or their combination. After growing for 2 weeks, formed clones were quantitated by using ImageJ. The synergistic anti-proliferative effect was evaluated by Combenefit. Blue indicates synergy, while red indicates antagonism between drugs.

(B) is representative of three biologically independent experiments. (E) represents mean ± SD of three independent experiments. P values were analyzed by Two-tailed unpaired Student’s t-test. Source data are provided as a Source Data file.

**Supplementary Figure 11**


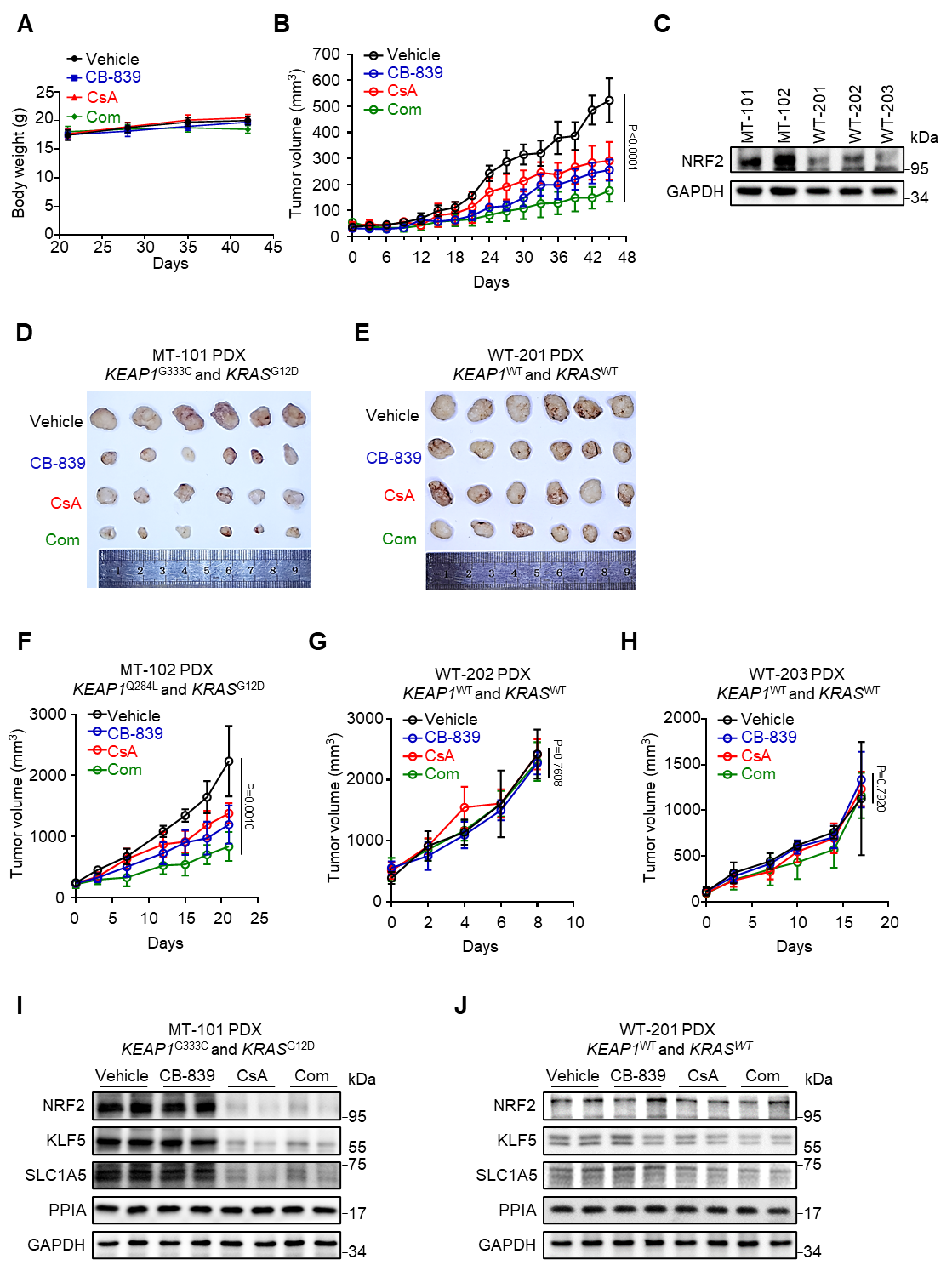


**Supplementary Figure 11. Related to Figure 6**

(A) Body weight of mice in lung orthotopic model formed by tail-vein injection of A549-luc cells (n=6 mice per group, **Figure 6C**). The body weight of each mouse was recorded weekly.

(B) Tumor growth in CD34^+^ HSC-derived humanized NCG mice bearing A549 xenograft tumors treated with the vehicle (p.o. twice daily), CB-839 (150 mg/kg, p.o. twice daily), CsA (20 mg/kg, i.p. every 3 days), and CB-839 and CsA drug combination (n = 5 for vehicle, CB-839 and CsA group; n = 4 for CB-839 and CsA drug combination group).

(C) Representative immunoblot analysis of NRF2 levels in MT-101, MT-102, WT-201, WT-202, and WT-203 PDX xenograft.

(D-E) Photographs of dissected PDX xenografts. D: MT-101 PDX; E: WT-201 PDX.

(F-H) Tumor growth in NCG mice bearing MT-102 (F), WT-202 (G) and WT-203 (H) PDX xenograft treated with the vehicle (PBS, p.o. twice daily), CB-839 (150 mg/kg, p.o. twice daily), CsA (20 mg/kg, i.p. every 3 days), and CB-839/CsA combination. MT-102 PDX harbors concurrent *KEAP1* and *KRAS* mutations *(KEAP1*^Q284L^ and KRAS^G12D^). WT-202 and WT-203 PDX has *KEAP1*^WT^ and *KRAS*^WT^. (n = 4 mice per group for F, n = 6 mice per group for G and H)

(I-J) Representative immunoblot analysis of NRF2, KLF5, SLC1A5 and PPIA levels in tumor tissues derived from MT-101 (Figure 6F) and WT-201 (Figure 6G) PDX xenografts.

The results of panels (C, I, J) are representative of three independent experiments. P values were analyzed using One-way ANOVA for (B) and (F-H). P < 0.05 was considered statistically significant. Source data are provided as a Source Data file.

**Supplementary Figure 12**


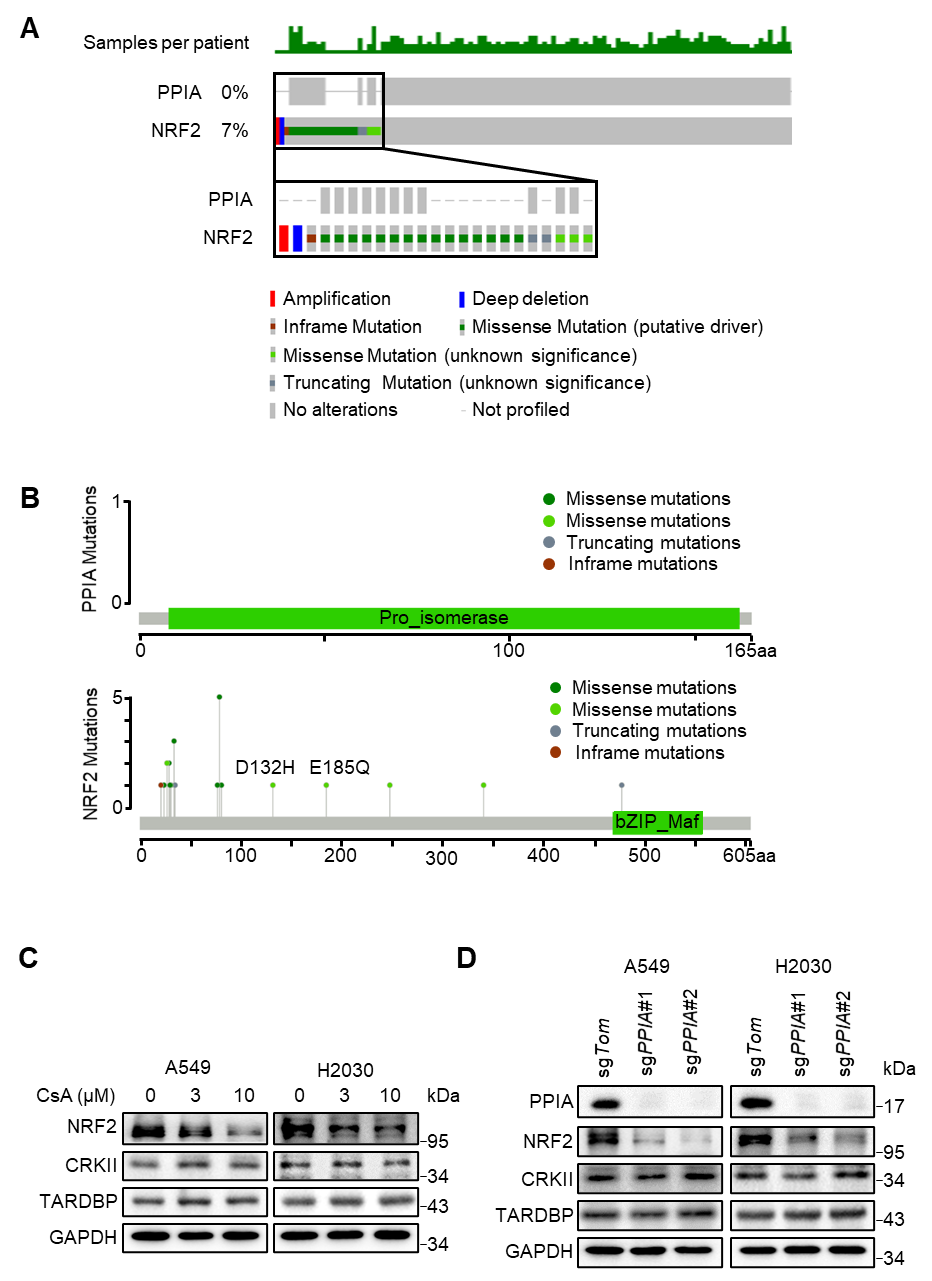


**Supplementary Figure 12. Related to Discussion**

(A) Gene alterations of PPIA and NRF2 in NSCLC cancer according to cBioPortal.

(B) Distribution of mutations in PPIA (upper) and NRF2 (lower) sequence coding area in NSCLC according to cBioPortal.

(C) Representative immunoblot analysis of CRKII and TARDBP levels in A549 and H2030 cells treated with CsA for 48 hours.

(D) Representative immunoblot analysis of CRKII and TARDBP levels in PPIA-WT or *PPIA*-KO A549 and H2030 cells.

The results of panels (C, D) are representative of three independent experiments. Source data are provided as a Source Data file.

**Supplementary Figure 13**

**
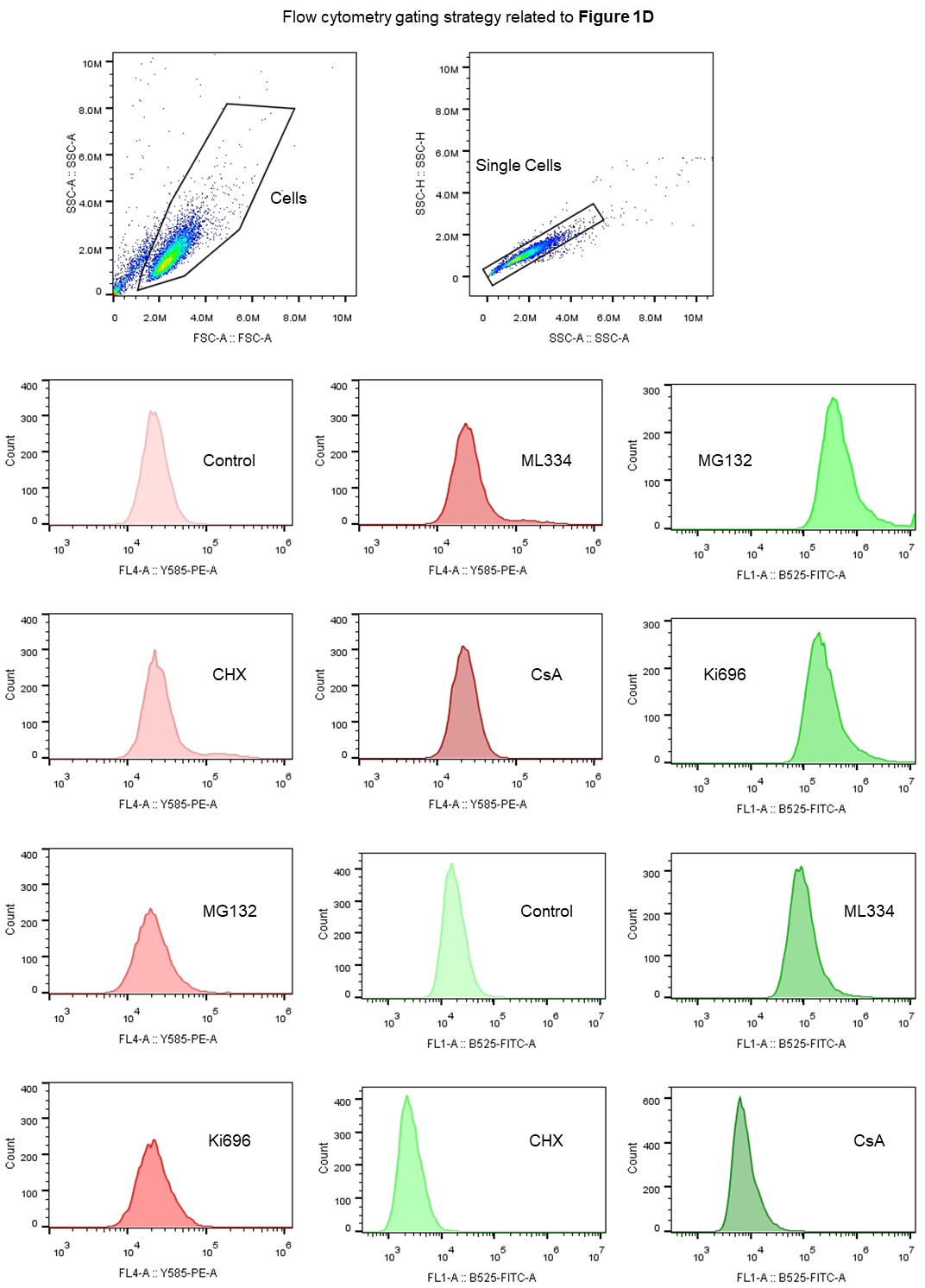
**

**Supplementary Figure 13. Flow cytometry gating strategy related to Figure 1D**

**Supplementary Table 1**

| **Cell** | **IC_50_ (μM) of CsA, n=3** |
| --- | --- |
| A549 | 1.24 ± 0.35 |
| H2030 | 1.58 ± 0.38 |
| H2122 | 1.57 ± 0.48 |
| H460 | 2.73 ± 0.49 |
| H441 | 3.39 ± 0.59 |
| H23 | 3.85 ± 0.12 |
| H838 | 4.40 ± 1.15 |
| H1299 | 6.22 ± 0.38 |
| H1703 | 8.18 ± 0.66 |
| H1395 | 8.92 ± 0.75 |
| H1975 | 9.47 ± 0.48 |
| H1650 | 12.67 ± 1.10 |
| H358 | 17.27 ± 1.56 |
| EBC-1 | > 20 |
| H1944 | > 20 |
| H292 | > 20 |
| H522 | > 20 |

**Supplementary Table 1:** IC_50_ values of CsA against 17 NSCLC cell lines related to Figure 1E.

**Supplementary Table 2**

| **Data statistics** | **PPIA/NRF2-PBM complex** |
| --- | --- |
| R work | 0.2336 |
| R free | 0.2478 |
| Resolution (Å) | 25.7-1.81 |
| NO. reflections | 15285 |
| Total number of atoms | 1304 |
| NO. protein residues | 165 |
| NO. water residues | 26 |
| F*o*, F*c* correlation | 0.94 |
| Bond length (Å) | 0.87 |
| Bond angles (°) | 1.03 |
| Favoured | 159 |
| Allowed | 4 |
| Outliers | 0 |

**Supplementary Table 2:** X-ray structure refinement statistics of PPIA/NRF2-PBM complex.

**Supplementary Table 3**

| **Gene Status** | | |
| --- | --- | --- |
| **PDX model** | **KEAP1** | **KRAS** |
| MT-101 | p.G333C | p.G12D |
| MT-102 | p.Q284L | p.G12D |
| WT-201 | NO. | NO. |
| WT-202 | NO. | NO. |
| WT-203 | NO. | NO. |

**Supplementary Table 3:** The *KEAP1* and *KRAS* gene mutation status of each PDX model.
