# Supplementary material for: PPIA dictates NRF2 stability to promote lung cancer progression
Source: Nat Commun. 2024 Jun 3;15:4703. doi: 10.1038/s41467-024-48364-4 (PMC11148020; doi:10.1038/s41467-024-48364-4)
Supplement: Supplementary file 2 — Description of Additional Supplementary Files [file 41467_2024_48364_MOESM2_ESM.pdf]

File name: Supplementary Data 1

Description: Primers for RT-PCR, siRNA, sgRNA, shRNA, ChIP and DNA sequencing used in this study.

File name: Supplementary Data 2

Description: The detail information of the antibodies, bacterial and virus strains, commercial regents, deposited data, cell lines, mouse strains, recombination DNA and software used in this study.

File name: Supplementary Data 3

Description: $^{13}$C-labeled glutamine flux analysis of A549 and *PPIA*-KO A549 cells.
